# Effect of thermal cycling on temperature changes and bond strength in different test specimens

**DOI:** 10.1080/26415275.2019.1709470

**Published:** 2020-01-29

**Authors:** Sigfus Thor Eliasson, Jon Einar Dahl

**Affiliations:** a Nordic Institute of Dental Materials, Oslo, Norway; b Faculty of Odontology, School of Health Sciences, University of Iceland, Reykjavik, Iceland

**Keywords:** Ageing, composite, dwell time, micro-tensile strength testing, shear strength testing, thermal gradient

## Abstract

**Objectives:**

To investigate temperature changes in various test specimens during thermal cycling and to evaluate difference in micro-tensile repair bond strength in specimens cut from the inner or the outer area of composite blocks after thermal cycling.

**Materials and methods:**

Four rectangular composite blocks of various sizes were fabricated, and thermocouples placed in the centre of the specimens or halfway from the surface to the centre. Composite cylinders were made on ground flat extracted molars, as intended for micro-tensile and shear bond testing, with a thermocouple placed at the centre of the cylinder radius between composite and dentin. The specimens were thermal cycled between 5 °C and 55 °C with 20–60 s dwell times. The highest and lowest temperatures in the test specimens were recorded.

Composite blocks were fabricated and stored in water for a week and then repaired with the same composite. After thermal cycling (5000×, 5 °C and 55 °C with a 20 s dwell time), test specimens were cut for micro-tensile testing.

**Results:**

None of the specimens tested reached the cold and warm water bath temperatures after a 20 s dwell time. In the smallest composite block, the centre core temperature reached 5 °C and 55 °C after 40 s dwell time, but lacked 1 °C after 60 s in the largest block. None of the specimens involving teeth reached water temperatures. The micro-tensile repair strength was significantly different between the outer and the central cut rods (*p* < .05).

**Conclusions:**

The most commonly used dwell times for thermal cycling are insufficient to create a homogeneous temperature change.

## Introduction

Well-controlled clinical studies are thought to be the ideal method for evaluating the success and longevity of dental restorative materials [1,2]. Unfortunately, clinical research on restorative materials is confounded by problems. It can take years to obtain any meaningful results in addition to be time consuming and costly with unpredictable patient dropout. Secondly, new materials are constantly introduced to the dental marked and, therefore, necessary to have methods to screen and evaluate new materials fast and effectively, and be able to estimate their pros and cons and possible clinical success. When testing or screening dental materials and bond strength to tooth structure in the laboratory, it is necessary to accelerate the simulated ageing process of the materials in the mouth, to be able to predict fastly the usefulness and the durability of the materials.

The most used methods for aging resin-based materials are storing in water and thermal cycling. Less clinically oriented and extreme aging procedures require boiling in water for 8 h and immersion in citric acid for a week [3,4]. Thermocycling is intended to simulate the thermal stress to which the restorative materials and the teeth would be exposed to by consuming drinks and food to get years of aging for the specimens in a short period of time [5]. Unfortunately, a standardized protocol for artificial aging of dental restorative materials does not exist, despite volumes of *in vitro* studies published and the fact that thermocycling is considered an inevitable method for ageing restorative materials [6]. Variations in the thermal cycling regimens are large in the literature and comparison of results is, therefore, often difficult [7]. Several investigators have measured the temperature fluctuations in the mouth when eating hot and cold food [8–12]. When reviewing the literature, it appears to be a general agreement among investigators that thermal cycling specimens between 5 °C and 55 °C is appropriate well to cover oral temperature fluctuations in the mouth [13]. Soh and Selwyn measured the temperature changes in the pulp chamber of molar teeth when thermocycled between 5 °C and 55 °C water baths with a 30 s dwell time [14]. They found 30 s to be insufficient time to reach water bath temperatures in the pulp chamber and longer dwell times to be needed. Fabris et al. used the finite elements method to simulate the effect of thermal stressing to porcelain fused with metal or zirconia. Their results indicated that the geometry of specimens significantly influenced the stresses generated and maximum stress to be located at the interface between the materials [15]. No studies could be found on the thermal stress developed nor on the thermal gradient within the various sized and shaped composite or tooth/composite specimen combinations during thermal cycling. It appears, however, from the literature that no agreement has been reached on dwell times and the number of cycles when specimens are thermocycled and that investigators determine these parameters on the bases of their convenience [13].

The purpose of this investigation is to measure temperature changes in various size and shaped test specimens at different dwell times during thermal cycling. In addition, to evaluate if there is difference in thermal stress in specimens taken from the inner and the outer parts of large composite blocks by studying repair bond strength after thermal cycling procedures according to the ISO/TS 11405 standard [16].

## Materials and methods

The restorative and dental materials used in this study are listed in Table 1.

**Table 1.** Materials used in the investigation.

Product	Manufacturer	Lot no.	Expiry date
Filtek™ Supreme XTE Universal Restorative A2B	3MEspe Dental Products, St. Paul, MN 55144-1000	N941996 N904514	2020-08 2020-11
Scotchbond Universal Adhesive	3MEspe Dental Products, St. Paul, MN 55144-1000	70831A	2019-07
Permadyne™Garant 2:1 Light bodied Consistency Polyether Impression Material	3MEspe Dental Products St. Paul, MN 55144-1000	3804066	2019-07

### Measurement of temperature changes in test specimens during thermal cycling

**Figure 1.**
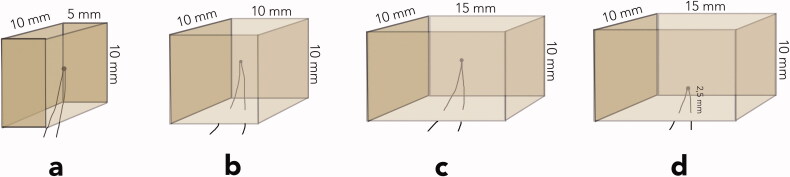
Various sized composite blocks, test specimens named a, b, c, and d with thermocouples placed in the centre or half-way from surface to centre.

Four rectangular composite blocks: specimen (a) 5 × 10 × 10 mm; specimen (b) 10 × 10 × 10 mm; specimens (c and d) 15 × 10 × 10 mm were fabricated in customized Teflon moulds. The composite blocks were incrementally built up with shade A2B Filtek Supreme XTE resin composite (3MEspe, St. Paul, MN) according to the instructions from the manufacturer, using a corded Demetron A2 led curing light (Kerr Corp., Orange, CA). The light output was measured at 1100 mW/cm^2^ (Norwegian Radiation Protection Authorities, Österaas, Norway). During the fabrication, thermocouples were placed in the centre of the specimens, except in specimen d, where the thermocouple was placed in the middle of and 2.5 mm above the largest surface (Figure 1).

Approximately, 75 cm long, thermocouple wires (fast response K type T010.0.20.1K.02000 thermocouple wires (Roth + Co. AG., Oberuzwil, Switzerland)) were connected to 80TK Thermocouple Module (Fluke Mfg. Inc., Ewerett, WA) and a digital thermometer (Escort EDM 168, Ter Calibration Ltd., Wigan, UK). The wires were 0.2 mm in diameter and isolated with fiberglass which was further protected by waterproof varnish. The thermocouples were calibrated at 0 °C ice water and in 50 °C Grant Type 100 thermal bath, with ±0.1 °C accuracy (Grant Instruments, Cambridge, UK) and further at 5 °C and 55 °C water baths against a certified thermometer at ±0.1 °C (Scalibra Calibration Lab., Skjetten, Norway).

Each specimen was placed in an automatic thermal cycling unit and transferred between thermostatically controlled 5 °C and 55 °C water baths, constantly stirred by electrically driven impellers. The size of each water bath was 8 l and the temperature in the digitally controlled 5 °C and 55 °C water baths was constantly verified using certified thermometers (Scalibra Calibration Lab., Skjetten, Norway). During thermal cycling, temperature fluctuations in the water baths were less than ±0.5 °C. The immersion or dwell time in each bath was for 20 s, 30 s, 40 s, and 60 s with a transfer time of 3 s. The thermal cycling unit was operated for approximately 10 cycles with specimens in place, or until temperature fluctuations had stabilized, before recording the highest and the lowest temperature in the specimens, 25 times for each parameter.

Two extracted non-restored human molars were used, taken from a biobank at NIOM with permission to be used for adhesive testing (2013/413 and 2014/457 approved by Regional Committees for Medical and Health Research Ethics, Norway).

**Figure 2.**
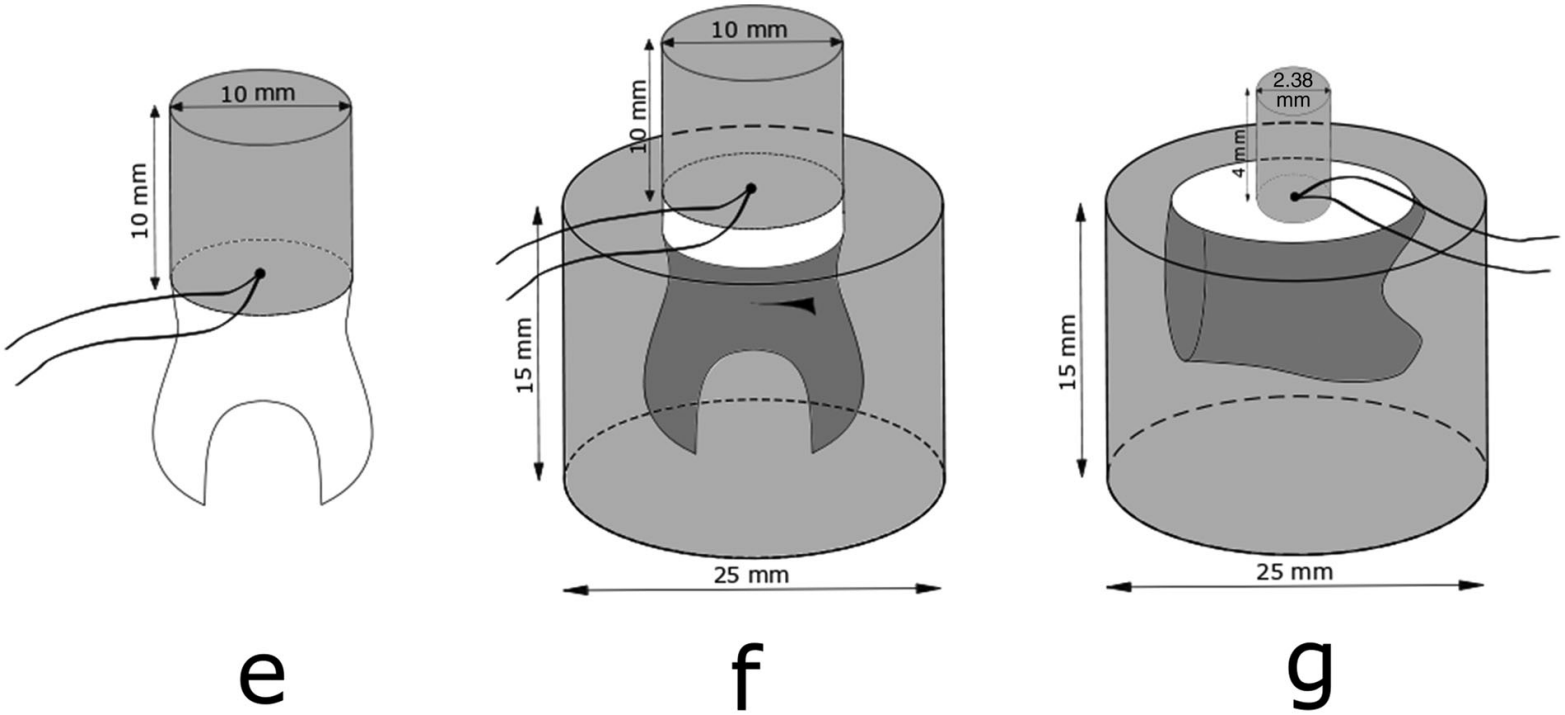
Test specimen assemblies named e, f, and g, with thermocouples placed in the centre radius between tooth and composite.

The occlusal third of one molar crown was cut off and ground flat with a 320-grit silicon carbide sandpaper disc (Struers, Copenhagen, Denmark) under running water to obtain a flat surface confined to superficial coronal dentin, as recommended by The Academy of Dental Materials and ISO/TS 11405 [16,17]. Using customized Teflon mould, the tooth was built up with Scotchbond Universal adhesive and shade A2B Filtek Supreme XTE resin composite (3MEspe, St. Paul, MN), resulting in a cylinder button measuring 10 mm in diameter and 10 mm in height, named specimen e. During the fabrication of the composite button, a thermocouple was placed in the centre of the cylinder radius between composite and dentin (specimen e, Figure 2). The tooth with the composite cylinder build-up was then thermal cycled the same way at the same dwell times and number of cycles as the composite blocks, and the highest and the lowest temperatures were recorded.

Next, the root portion of the tooth specimen was placed in a 25 mm in diameter and 15 mm high mould, which was filled with self-curing resin, and after that procedure, named specimen f (Figure 2). The tooth/composite assembly with the root mounted in resin was then thermal cycled the same way as before and the highest and the lowest temperatures were recorded 25 times.

The root was cut off the second molar and the mesial crown surface ground flat into the dentin and the tooth mounted in a resin block, 25 mm in diameter and 15 mm high, according to ISO 29022 for Notched – edge shear bond strength test [18]. After further grounding the tooth surface flat with a 320-grit silicon carbide sandpaper disc (Struers, Copenhagen, Denmark) under running water, the tooth was built up, using customized Teflon mould and same materials, Scotchbond Universal adhesive and shade A2B Filtek Supreme XTE resin composite (3MEspe, St. Paul, MN), but now resulting in a cylinder button measuring 2.38 mm in diameter and 4 mm high (specimen g). During the fabrication of the composite button, a thermocouple was placed in the centre of the cylinder radius between composite and dentin (Figure 2). The assembly was then thermal cycled the same way as before and the highest and the lowest temperatures were recorded 25 times.

The position of all thermocouples was verified by taking X-rays of the composite blocks and tooth specimen assemblies. While thermal cycling the specimens, all digital thermometer readout was video recorded, and high and low temperatures verified when played at a low speed.

### Micro-tensile repair bond strength measurements of specimen rods obtained from outer and centre areas of large thermal cycled composite blocks

Eight, shade A2B Filtek Supreme XLT composite blocks (3MEspe, St. Paul, MN), 10.5 mm × 10.5 mm wide and 8 mm high, were fabricated in a Teflon mould in accordance to the instructions from the manufacturer. The composite blocks were incrementally built in four layers and each layer cured with a corded Demetron A2 LED curing light (Kerr Corp., Orange, CA) for 40 s on five overlapping areas, each corner of the mould and in the centre of the specimen. The specimens were not further cured upon removal from the mould. The light output was measured at 1100 mW/cm^2^ (Norwegian Radiation Protection Authorities, Österaas, Norway). Mylar strip and glass slides were used at both ends of the Teflon mould to achieve flat-ended specimen blocks.

After polymerisation, the composite blocks were immediately stored in distilled water and aged for a total of one week. The test surface was then ground on a 320-grit silicon carbide sandpaper (Struers, Copenhagen, Denmark) under running water for 5 s to obtain a flat surface with standardized roughness. For cleaning purposes, the test surfaces of all the experimental composite blocks were acid etched with 37% phosphoric gel for 15 s and rinsed with water for another 15 s and dried for 5 s with oil-free air stream.

Scotchbond Universal Adhesive (3MEspe, St. Paul, MN), a one-step self-etching adhesive, was applied and cured according to the recommendations from the manufacturers. After surface treatment and adhesive application, the original mould was placed over the aged composite blocks and the first repair composite layer placed. The aged composite blocks were repaired in approximately 2 mm incremental layers with the same composite material as the original aged specimen blocks, using two consecutive 5 mm Teflon moulds with guided pins, resulting in 18 mm high specimens. After this, the composite blocks were placed in distilled water for 3 months. Then six of the specimen blocks were thermal cycled 5000 times between 5 °C and 55 °C with 20 s dwell time as recommended in ISO/TS 11405 [16] and 3 s transfer time. The two remaining specimen blocks were not thermal cycled and used as a control group.

**Figure 3.**
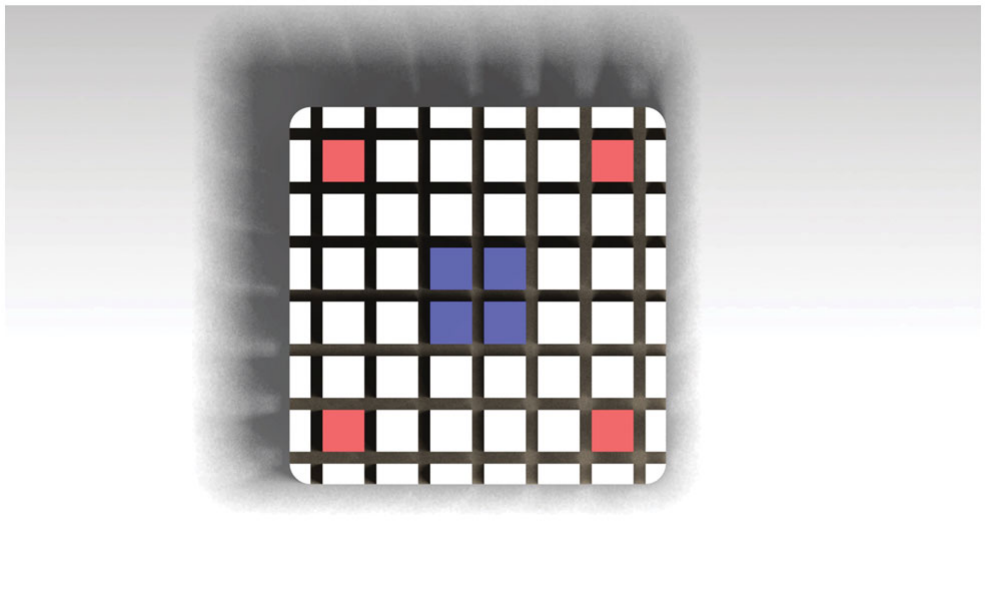
Overview of composite block cut in the *x* and *y* directions, with rod ends color-coded for identification of position and used for micro-tensile testing.

The composite blocks were mounted on an automatic cutting machine (Metcon®, Miracut 201 Automatic Precision Cutter, Bursa, Turkey) equipped with a water-cooled thin diamond blade. The specimens were serially sectioned perpendicular to the bonding surface, both in the *x* and the *y*-axis, producing number of square test specimen rods approximately 1.1 × 1.1 mm. After the first cuts, light-bodied impression material (Permadyne Garant 2:1, 3MEspe Dental Products, MN) was injected into the cuts for support before the second cuts. Thirty-six specimen rods were obtained from each composite cylinder. The rods were colour coded at the ends for the identification of the position in the specimen (Figure 3). Only four outer corner specimen rods (red) and four inner centre rods (blue) were used for testing. The test specimens were cleaned ultrasonically in distilled water for 3 min. After the cleaning procedure, the test specimen rods were examined light microscopically at a magnification of 40× for voids and imperfections (Nexius Zoom, Euromex, Netherlands). The width and the thickness of each test specimen were measured to the nearest 0.01 mm using a calibrated digital calibre (Mitutoyo Co, Kawasaki, Japan).

Our recently published and less time-consuming method was used for the tensile testing [19,20]. Each test specimen was mounted in a calibrated Universal testing machine (Lloyd Instruments LTD, Model LRX, Fareham, England), using specially attached steel wires designed to transmit pure tensile forces to the specimen. The testing was conducted at a crosshead speed of 1 mm/min until fracture. The tensile bond strength of each test specimen was calculated in MPa, by dividing the imposed force (in Newton’s) at fracture by the cross-sectional bond area (in mm^2^). All test specimens were maintained moist throughout the preparation and the test procedure.

Statistical calculations were according to suggestions in the ISO/TS 11405:2015 Technical Specification for the treatment of results for testing of adhesion [16].

## Results

The results for the temperature changes are presented in Tables 2–5. In general, it appears that the specimens reach or approach the water bath temperature earlier in the warm bath than in the cold bath. At a 20 s dwell time, none of the composite block specimens tested reached the 50 °C uniform temperature difference in the centre of the specimens. At a 20 s dwell time, the temperature difference was only 44.4 °C in the smallest specimen and 24.7 °C in the largest one. In the smallest composite block, specimen a, the centre core temperature reached 5 °C and 55 °C after 40 s dwell time. It lacks less than 1 °C for the largest block, specimen c, to reach 5 °C and 55 °C at the centre after 60 s dwell time. In specimen d, which is the same size as specimen c, except thermocouple placed at 2.5 mm depth, the temperature had reached 55 °C at 50 s in the hot bath, but 5.4 °C at 60 s in the cold bath. None of the specimens involving teeth reached the water temperatures in the centre of the cylinders at the composite dentin interface at the dwell times used. At a 20 s dwell time, the temperature difference was only 24.6 °C in specimen e, 14.4 °C in specimen f, and 45.6 °C in specimen g. At 60 s dwell time, temperatures measured in specimen g were closest to water bath temperatures, 6.0 °C in the cold bath and 54.7 °C in the warm bath but only 12.9 °C and 46.9 °C in specimen f, respectively.

**Table 2.** Temperature measured in resin based composite test specimens in 5 °C water bath at different dwell times (*n* = 25).

	Specimen designation			
Specimen size	a 5 x 10 x 10 mm	b 10 x 10 x 10 mm	c 15 x 10 x 10 mm	d 15 x 10 x 10 mm
Thermocouple	Centre	Centre	Centre	2.5 mm from surface
Dwell time (s)				
20	7.8 ± 0.11 °C	14.3 ± 0.25 °C	17.7 ± 0.22 °C	12.3 ± 0.65 °C
30	5.6 ± 0.06 °C	9.2 ± 0.09 °C	12.1 ± 0.13 °C	8.7 ± 0.19 °C
40	5.2 ± 0.11 °C	6.3 ± 0.15 °C	8.0 ± 0.18 °C	6.6 ± 0.16 °C
50	5.0 ± 0.05 °C	5.7 ± 0.16 °C	6.8 ± 0.11 °C	6.1 ± 0.13 °C
60	5.0 ± 0.06 °C	5.3 ± 0.15 °C	5.7 ± 0.07 °C	5.4 ± 0.16 °C

**Table 3.** Temperature measured in resin based composite test specimens in 55 °C water bath at different dwell times (*n* = 25).

	Specimen designation			
Specimen size	a 5 x 10 x 10 mm	b 10 x 10 x 10 mm	c 15 x 10 x 10 mm	d 15 x 10 x 10 mm
Thermocouple	Centre	Centre	Centre	2.5 mm from surface
Dwell time (s)				
20	52.2 ± 0.14 °C	45.2 ± 0.37 °C	42.4 ± 0.44 °C	48.0 ± 1.01 °C
30	54.3 ± 0.08 °C	50.4 ± 0.20 °C	48.0 ± 0.14 °C	51.4 ± 0.10 °C
40	55.2 ± 0.02 °C	53.0 ± 0.63 °C	51.1 ± 0.23 °C	53.4 ± 0.14 °C
50	55.2 ± 0.11 °C	54.4 ± 0.17 °C	53.1 ± 0.32 °C	55.2 ± 0.14 °C
60	55.2 ± 0.14 °C	55.0 ± 0.07 °C	54.2 ± 0.10 °C	55.1 ± 0.17 °C

**Table 4.** Temperature measured in the centre of specimens at the adhesive layer in 5 °C water bath at different dwell times (*n* = 25).

	Specimen designation		
	e	f	g
Specimen size	10 mm Ø×10 mm composite cylinder on tooth	10 mm Ø×10 mm composite cylinder on tooth, mounted in resin	2.38 mm Ø×4 mm composite cylinder on tooth, mounted in resin
Dwell time (s)			
20	17.6 ± 0.35 °C	22.4 ± 1.40 °C	7.6 ± 0.13 °C
30	13.0 ± 0.15 °C	18.5 ± 0.19 °C	7.0 ± 0.08 °C
40	8.7 ± 0.31 °C	16.0 ± 0.56 °C	6.2 ± 0.15 °C
50	7.4 ± 0.27 °C	14.3 ± 0.25 °C	6.1 ± 0.19 °C
60	6.3 ± 0.09 °C	12.9 ± 0.17 °C	6.0 ± 0.14 °C

**Table 5.** Temperature measured in the centre of specimens at the adhesive layer in 55 °C water bath at different dwell times (*n* = 25).

	Specimen designation		
	e	f	g
Specimen size	10 mm Ø×10 mm composite cylinder on tooth	10 mm Ø×10 mm composite cylinder on tooth, mounted in resin	2.38 mm Ø×4 mm composite cylinder on tooth, mounted in resin
Dwell time (s)			
20	42.2 ± 0.45 °C	37.8 ± 1.00 °C	53.2 ± 0.15 °C
30	46.9 ± 0.20 °C	41.6 ± 0.17 °C	53.9 ± 0.14 °C
40	50.2 ± 0.16 °C	43.7 ± 0.66 °C	54.2 ± 0.28 °C
50	52.4 ± 0.30 °C	45.8 ± 0.19 °C	54.4 ± 0.20 °C
60	53.8 ± 0.22 °C	46.9 ± 0.12 °C	54.7 ± 0.15 °C

The results for the micro-tensile repair bond testing are presented in Table 6. Twenty-four specimens were obtained for each test group and eight for each control test group. The mean tensile strength in the thermal cycled group was 37.5 ± 8.8 MPa for the outer corner specimen group and 55.5 ± 8.5 MPa for the inner central specimen group (*p* < .05). The mean tensile strength was 58.3 ± 4.2 MPa for the outer corner specimens and 59.1 ± 6.4 MPa for the inner central specimens in the control group. There was no statistical difference between the inner thermal cycled group and the inner and outer control groups, respectively.

**Table 6.** Repair micro-tensile bond strength of test specimens stored in water for 3 months and tested after thermal cycling and without thermal cycling.

3 months in water + thermal cycling (*n* = 24 in each group)		3 months in water (control) (*n* = 8 in each group)	
outer specimens	inner specimens	outer specimens	inner specimens
37.8 ± 8.8 MPa^A,B,C^	55.5 ± 8.5 MPa^A^	58.3 ± 4.2 MPa^B^	59.1 ± 6.4 MPa^C^

Groups with same upper-case letters are statistically significant different (*p* < .05).

## Discussion

There is no generally accepted protocol for resin composites that simulates and accelerates ageing of the materials under oral conditions. Even though thermal cycling is one of the procedures used to seriously try to simulate physiological aging of restorative materials, review of the literature on thermal cycling procedures shows that there is little or no consistency in protocols and variables. This makes it difficult to compare results from the various investigations. Selection of variables like size of specimens, storage time, and dwell times in water baths, transfer time and number of cycles, appear to be selected by investigators mostly based on convenience rather than referenced observations or scientifically founded facts [7,13]. The water bath temperatures used, appear to have scientifically better ground [8,10,21]. Palmer recorded 1 °C temperature when biting on ice cube [10] and Peterson reported that 10 °C to be tolerable but 15 °C was without discomfort [5]. He measured lower anterior incisor which could have been more sensitive to cold than the molar tooth used by Palmer [7]. The same applies for the highest tolerable temperature where Plant et al. found that coffee was too hot to drink at 68 °C, could be drunk with discomfort between 60 °C and 68 °C, but could be drunk in large amounts between 50 °C and 55 °C, despite it was considered relatively hot [22].

Although some variation in temperature changes measured in the mouth and different tolerance to extreme temperatures have been reported, there appears to be a general agreement among investigators on the temperature gradient from 5 °C to 55 °C, when thermal cycling specimens in laboratory testing [7,13]. These temperatures are also in accordance with ISO TS 11405 Technical Specification for testing of adhesion to tooth structure [16].

When consuming hot and cold, another important parameter than temperature must also be considered: volume of fluid or food taken into the mouth as well as time of contact with teeth, which is usually only for few seconds [21]. Amaral suggested that people would not tolerate extreme hot and cold substances in contact with teeth for extended time period [23]. Also, in a recent *in vivo* study, it was suggested that the maximum exposure time to tooth surface of extreme temperatures was only 2–5 s [24]. As a result, several authors in their studies have used these short ‘clinically oriented’ times as dwell times when thermal cycling [25–27]. While these short dwell times could possibly be used e.g. in leakage studies or other studies where restored teeth are thermal cycled, our results show that much longer dwell times are needed for the temperature gradient to reach evenly throughout the specimen. When large specimens are to be cut into smaller pieces or rods after thermal cycling, like for micro-tensile testing, the thermal stress must reach evenly throughout the entire interphase. Therefore, short exposure or dwell times to the different temperatures experienced in the oral cavity, does not apply in such situations.

The number of cycles in laboratory testing has not been based on scientific data, but rather on opinion or estimation. Lloyd et al. reported on similarity in enamel cracks *in vivo* after several years of service, and in newly erupted extracted teeth thermal cycled several thousand times (4000×) [28]. Gale and Darwell suggested in their review paper very complicated thermal cycling procedure with 10,000 cycles and sequence of temperatures: 35 °C, 15 °C, 35 °C, and then 45 °C with corresponding dwell times of 28 s, 2 s, 28 s, and 2 s [7]. This was supposed to represent natural variability *in vivo*. We could not find any study using this method. Youngson and Barclay vent even further and suggested using six different water bath temperatures [11]. It is important to agree on the number of cycles that are reasonably many and can be accepted by most researchers like 5000 cycles, as used in this and numerous other investigations.

The various sizes of specimens and dwell times used in this investigation revealed some interesting and logic observations. The total mass of the composite blocks played an important role with the thermal gradient being slower in larger specimens. Low thermal conductivity and thermal diffusivity of resin composites further contributes to slow heat transfer to the centre of the specimens [29]. When thermal cycling the various size composite blocks, both hot and cold water temperatures were not reached after the most commonly used dwell times (20–30 s) [7,17,30] and for the larger specimens, water bath temperatures were not quite reached after 60 s. It must also be noted that temperature fluctuations in both water baths were less than ±0.5 °C, and the standard deviation of temperature measurements in the test specimens was negligible. In other words, when temperature was reached during thermal cycling, both high and low temperatures recorded were stable for all the sizes and shapes of specimens.

Tooth specimen g (Figure 2) is prepared according to ISO 29022 International Standard for Notched – edge shear bond strength testing [18]. There, the composite button is only 2.38 mm in diameter, bonded to a tooth mounted in a resin cylinder. When that specimen assembly was thermal cycled between 5 °C and 55 °C, the centre point temperature between tooth and this small cylinder does not reach the water temperatures at the most popular 20–30 s dwell times used in numerous research projects [7,17], and even not quite at 60 s dwell time. These results further indicate that the mass of the specimen assembly plays a major role when it is aged *in vitro* by thermal cycling. This assumption is supported by Soh and Selwyn, who concluded that dwell times needed to reach the water bath temperatures in pulp chamber of teeth depended on the thickness of dentin [14]. For this type of shear bond testing, reducing the resin block that the tooth is mounted into, e.g. 15 mm in diameter and 10 mm high, would most likely be sufficient for the 5 °C and 55 °C temperature gradient to penetrate throughout the adhesive interface when thermal cycling with 60 s dwell time. It must, however, be recognized that thermal cycling is of no value if the initial bond at the interface between two materials is not already known to have basic strength or reliability [7]. It is important to avoid both intense and low stress when determining values for simulating physiological aging of biomaterials like resin composites. Too extreme temperatures could possibly inadvertently alter the properties of the materials to be investigated, while too little stressing could lead to that inefficient materials would be accepted into clinical use [10,13].

Tooth specimens e and f in Figure 2 are fabricated according to ISO/TS 11405 and as suggested in the guidance from the Academy of Dental Materials for *in vitro* micro-tensile bond strength testing of dental composite bonding effectiveness to dentin [16,17]. In the guidance, it is stated that ‘thermo-mechanical ageing was best performed in the macro-specimen form’. Our results show that the centre temperature in the interphase between tooth and the composite cylinder is far from reaching the water bath temperatures when thermal cycled with 20 s dwell time. The same applies even though the dwell time is increased to 60 s, being even more obvious when the tooth is mounted in the resin cylinder as recommended [17]. It must be recognized that the tooth used was large and it is unusual to utilize as large composite cylinder button, both in radius and height. Reducing the mass of the tooth/composite macro-specimen assembly, e.g. by reducing root ends and resin cylinder for tooth mounting and limiting the size of composite build-up, would help the temperature gradient to reach throughout the specimen and thereby uniformly stress the adhesive interface.

The results of the second part of this investigation revealed that the micro-tensile repair strength of rods taken from the outer part of large specimens were lower than that of rods taken from the inner part after thermal cycling. It can be assumed that the inner and the outer part of a large composite block received different stresses during thermal cycling due to temperature gradient in the material. The temperature measurements in composite block of similar size verified this. The 20 s dwell time used in the present experiment was the minimum time recommended in the guidance from the Academy of Dental Materials and has been used in numerous similar investigations [7,13,17]. Our results could in part explain the high standard deviation frequently reported in micro-tensile adhesion studies, when thermal cycling was used for ageing specimens prior to cutting into the smaller test specimens [31–33]. Considering the results from our investigation, it might be advantageous to cut the macro specimens into rods before thermal cycling or increase the dwell time to obtain a homogeneous temperature in the test specimens.

## Conclusions

The temperature changes in test specimens during thermal cycling between 5 °C and 55 °C water baths are largely dependent on specimen assembly size or total mass and dwell time in the water baths. The most common dwell times, 20–30 s used for thermal cycling, are for most test specimens insufficient. Therefore, the test on thermal cycling given in ISO/TS 11405 [16] is regarded as inadequate and an amendment is necessary.

Suggested universal thermal cycling protocol for macro specimens is:

Temperature gradient: 5 °C and 55 °C.

Number of cycles: 5000 cycles.

Dwell time: 60 s.

Transfer time: short.
